# Comparison of cardiovascular magnetic resonance characteristics and clinical consequences in children and adolescents with isolated left ventricular non-compaction with and without late gadolinium enhancement

**DOI:** 10.1186/s12968-015-0148-7

**Published:** 2015-05-30

**Authors:** Huaibing Cheng, Minjie Lu, Cuihong Hou, Xuhua Chen, Li Li, Jing Wang, Gang Yin, Xiuyu Chen, Wei Xiangli, Chen Cui, Jianmin Chu, Shu Zhang, Sanjay K Prasad, Jielin Pu, Shihua Zhao

**Affiliations:** Center for Arrhythmia Diagnosis and Treatment, State Key Laboratory of Cardiovascular Disease, Fuwai Hospital, National Center for Cardiovascular Diseases, Chinese Academy of Medical Sciences and Peking Union Medical College, 100037 Beijing, People’s Republic of China; Department of Radiology, State Key Laboratory of Cardiovascular Disease, Fuwai Hospital, National Center for Cardiovascular Diseases, Chinese Academy of Medical Sciences and Peking Union Medical College, 100037 Beijing, People’s Republic of China; Department of Pathology, State Key Laboratory of Cardiovascular Disease, Fuwai Hospital, National Center for Cardiovascular Diseases, Chinese Academy of Medical Sciences and Peking Union Medical College, 100037 Beijing, People’s Republic of China; Cardiovascular Magnetic Resonance Unit, NIHR Biomedical Research Unit, Royal Brompton Hospital, Sydney Street, SW3 6NP London, UK

**Keywords:** Left ventricular non-compaction, Cardiovascular magnetic resonance, Late gadolinium enhancement, Children

## Abstract

**Background:**

Although cardiovascular magnetic resonance (CMR) is showing increasingly diagnostic potential in left ventricular non-compaction (LVNC), relatively little research relevant to CMR is conducted in children with LVNC. This study was performed to characterize and compare CMR features and clinical outcomes in children with LVNC with and without late gadolinium enhancement (LGE).

**Methods:**

A cohort of 40 consecutive children (age, 13.7 ± 3.3 years; 29 boys and 11 girls) with isolated LVNC underwent a baseline CMR scan with subsequent clinical follow-up. Short-axis cine images were used to calculate left ventricular (LV) ejection fraction (EF), end-diastolic volume (EDV), end-systolic volume (ESV), myocardial mass, ratio of non-compacted-to-compacted myocardial thickness (NC/C ratio), and number of non-compacted segments. The LGE images were analyzed to assess visually presence and patterns of LGE. The primary end point was a composite of cardiac death and heart transplantation.

**Results:**

The LGE was present in 10 (25 %) children, and 46 (27 %) segments were involved, including 23 non-compacted segments and 23 normal segments. Compared with LGE- cohort, LGE+ cohort had significantly lower LVEF (23.8 ± 10.7 % vs. 42.9 ± 16.7 %, *p* < 0.001) and greater LVEDV (169.2 ± 65.1 vs. 118.2 ± 48.9 mL/m^2^, p = 0.010), LVESV (131.3 ± 55.5 vs. 73.3 ± 46.7 mL/m^2^, *p* = 0.002), and sphericity indices (0.75 ± 0.19 vs. 0.60 ± 0.20, *p* = 0.045). There were no differences in terms of number and distribution of non-compacted segments, NC/C ratio, and myocardial mass index between LGE+ and LGE- cohort. In the LGE+ cohort, adverse events occurred in 6 patients compared to 2 events in the LGE- cohort. Kaplan-Meier analysis showed a significant difference in outcome between LGE+ and LGE- cohort for cardiac death and heart transplantation (*p* = 0.011).

**Conclusions:**

The LGE was present in up to one-fourth of children with LVNC, and the LGE+ children exhibited a more maladaptive LV remodeling and a higher incidence of cardiovascular death and heart transplantation.

## Background

Left ventricular non-compaction (LVNC) is a genetically and clinically heterogeneous cardiomyopathy characterized by numerous prominent trabeculations, progressive myocardial dysfunction, malignant ventricular arrhythmias, and early mortality [1, 2]. Left ventricular non-compaction can present in isolation or in combination with other congenital heart diseases or genetic neuromuscular conditions [3–5]. Previous work has shown that LVNC accounts for about 9 % of cardiomyopathy in childhood [6]. The incidence of isolated LVNC diagnosed at children echocardiograms was estimated to be approximately 0.2 % [7].

Cardiovascular magnetic resonance (CMR) may outperform echocardiography in assessing the whole left ventricular (LV) myocardium and also provide precise identification of prominent trabeculations owing to its higher spatial resolution and field of view [8–10]. Furthermore, CMR with late gadolinium enhancement (LGE) is a reliable technique for detecting myocardial fibrosis in vivo, which is related to clinical severity grading and prognosis in adult patients with LVNC [11–15]. Although CMR is showing increasingly diagnostic potential in LVNC, works on the application of CMR in children with LVNC are restricted to several case reports and small case series [16–19]. Therefore, we have assembled a relatively large cohort of children with LVNC, defined by eligibility for CMR, to characterize and compare CMR features and clinical outcomes in children with LVNC with and without LGE.

## Methods

### Study patients

We prospectively recruited a cohort of consecutive children with isolated LVNC who were referred to the Fuwai Hospital for CMR between June 2006 and December 2013. The diagnosis of LVNC was made on the basis of previously defined CMR and clinical criteria: [9] (a) appearance of 2 distinct myocardial layers; (b) prominent myocardial trabeculations and deep intertrabecular recesses communicating with the LV cavity; (c) end-diastolic ratio of non-compacted-to-compacted (NC:C) myocardium >2.3:1, and (d) absence of other known co-existing cardiac abnormalities. The study complied with the Declaration of Helsinki and was approved by the Fuwai Hospital ethics committee, and informed consent was obtained from the parents of each child with LVNC.

### CMR image acquisition and analysis

All CMR exams were performed using a 1.5-T scanner (Siemens Avanto). Retrospective electrocardiographic gated cine images were performed using true fast imaging with steady-state free precession sequence (image parameters: repetition time/echo time [TR/TE] = 40.0/1.1 ms; matrix = 256 × 192 mm; flip angle = 62°) in three long-axis (horizontal and vertical long-axis and LV outflow tract) and continuous short-axis views covering the entire LV from base to apex. Fifteen ± 5 min after the injection of 0.2 mmol/kg of gadolinium-DTPA (Magnevist; Schering, Berlin, Germany), the LGE images were obtained using an inversion recovery sequence (image parameters: TR/TE = 8.7/3.4 ms, matrix = 256 × 256 mm, flip angle = 15°) in three long-axis and standard short-axis views covering the whole LV.

**Fig. 1:**
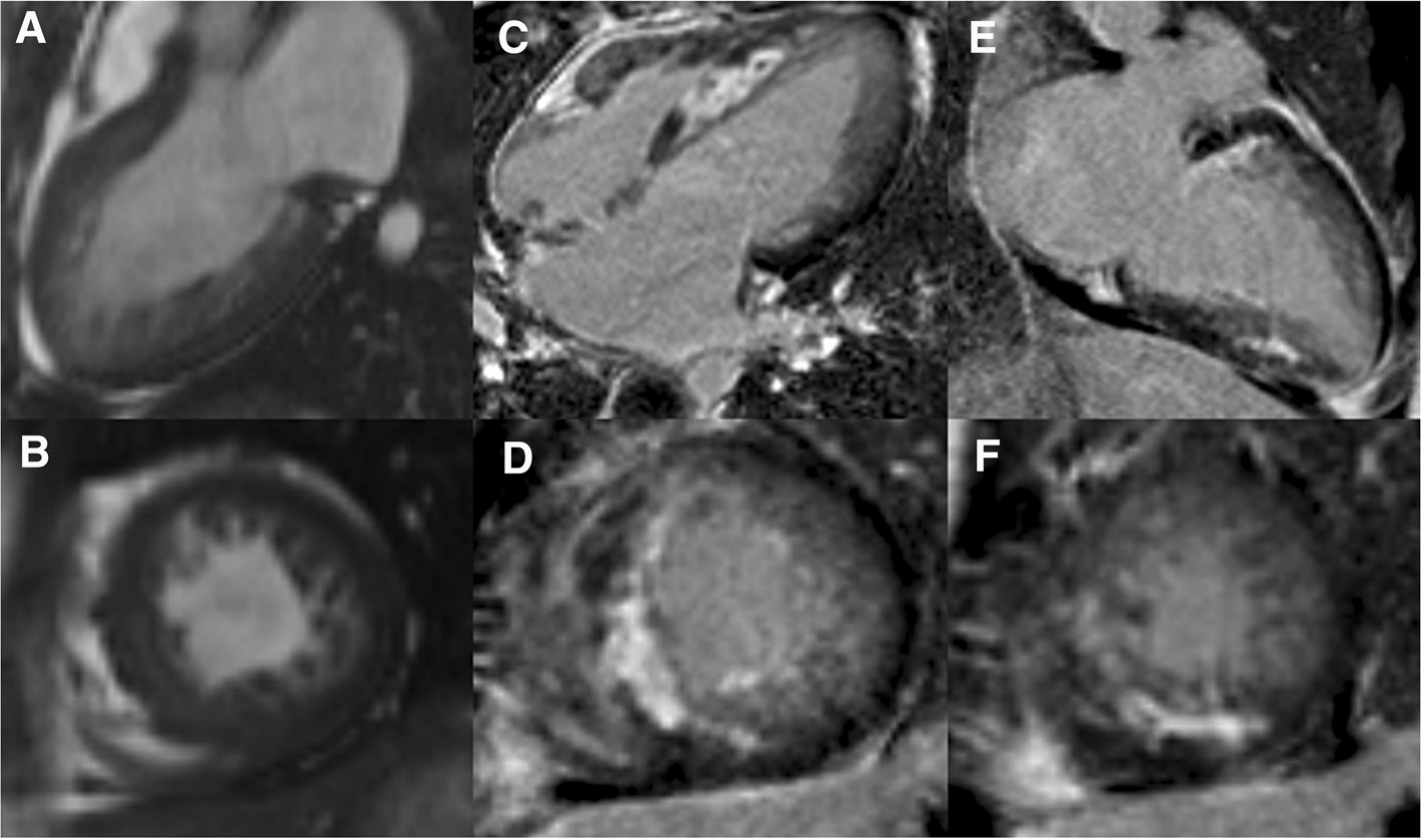
Representative images of LGE in a 16-year-old boy with LVNC. The vertical (**a**) and horizontal (**c**) long-axis and short-axis (**b** and **d**) contrast-enhanced images demonstrate transmural LGE in the septal and inferior segments

All images were analyzed using a workstation with commercially available software (Siemens Argus). For children with multiple CMR studies, the initial baseline study was used for the primary analysis. Results for LV ejection fraction, ventricular volumes, and myocardial mass were derived from short-axis slices. As previously demonstrated, the papillary muscles were excluded from compacted myocardium because it is difficult to differentiate papillary muscles from dense trabeculations [20]. Left ventricular volumes and compacted mass were indexed to body surface area. The LV sphericity index was calculated as: end-diastolic volume (EDV)/([end-diastolic long-axis diameter^3^ × π]/6) [21]. The presence or absence of non-compaction and LGE was qualitatively assessed using the AHA 17 segment model [22]. For each segment with non-compaction, the end-diastole NC/C ratio was quantitatively calculated in the short-axis views, and the maximum ratio was then used for analysis. As previously demonstrated, the assessment of NC/C ratio of the apex (segment 17) was excluded [9]. LGE was deemed present only if myocardial enhancement was confirmed on both short-axis and matching long-axis areas using a signal intensity threshold of ≥ 6 standard deviations (SD) above a remote reference region (Fig. 1). The myocardial layer distributions of LGE were assessed using the following scale: 1 = subepicardial, 2 = mid-wall, 3 = subendocardial, and 4 = transmural (≥75 % LV wall thickness). The LGE score was finally summed. The evaluations of non-compaction and LGE were performed by 2 independent expert readers (ML and HC) who were blinded to the clinical data. Discordant findings were resolved by consensus.

### Follow-up

All children were followed up via clinic visits or telephone interview after initial CMR examination. The endpoint of the study was cardiovascular death and heart transplantation. The duration of follow-up was determined from the first CMR evaluation date to the occurrence of an endpoint. Complete follow-up was available for all children.

### Statistical analysis

Normality was tested using the Kolmogorov-Smirnov test. Continuous variables were expressed as mean ± SD and assessed using unpaired t tests or Wilcoxon’s rank-sum tests. Categorical variables were expressed as frequencies (percentages) and assessed using the *χ*^2^ or Fisher exact tests. Correlation analyses were performed using the Spearman’s rank correlation test. Survival curve was generated by the Kaplan-Meier method and compared by the log rank test. The statistical analysis was performed using SPSS for Windows 16.0 (Chicago, IL, USA). A two tailed p value of < 0.05 was considered statistically significant.

## Results

### Baseline characteristics

A cohort of 40 children was diagnosed with isolated LVNC. An additional 8 children with prominent trabeculations who met CMR diagnostic criteria were associated with either congenital heart diseases and thus excluded from the study, such as atrial septal defect (n = 4), Ebstein’s anomaly (n = 1), aorto-left ventricular tunnel (n = 1), membranous subaortic stenosis (n = 1), bicuspid aortic valve (n = 1). Twenty-nine children (72 %) were male and 11 (28 %) were female. The mean age at the time of initial CMR scan was 13.7 ± 3.3 years (range, 1.5-17 years). Three children (8 %) had a family history of LVNC. Twenty-one children (53 %) presented primarily with signs and symptoms of congestive heart failure. Seven children (18 %) presented with documented ventricular arrhythmias and 1 patient (3 %) presented with aborted sudden death. Five children (13 %) were referred for evaluation of unexplained syncope. Two children (5 %) presented with a primary complaint of chest pain. No children had signs of systemic emboli. Clinical and demographic characteristics of the study population are summarized in Table 1.

**Table 1:** Baseline characteristics

	All Children	Presence of LGE		
Variable	(n = 40)	Yes (n = 10)	No (n = 30)	p Value
Age (years)	13.7 ± 3.3	14.0 ± 2.1	13.6 ± 3.6	0.753
Male, n (%)	29 (73)	9 (90)	20 (67)	0.233
Family history of LVNC, n (%)	3 (8)	1 (10)	2 (7)	1.000
Symptoms				
Dyspnoea, n (%)	21 (53)	9 (90)	12 (40)	0.004
Chest pain, n (%)	2 (5)	0	2 (7)	1.000
Syncope/pre-syncope, n (%)	7 (18)	1 (10)	6 (20)	0.651
Thrombo-embolic events, n (%)	0	0	0	
NYHA functional class	2.4 ± 1.0	3.0 ± 0.9	2.1 ± 0.9	0.016
I, n (%)	10 (25)	1 (9)	9 (30)	
II, n (%)	11 (28)	1 (9)	10 (33)	
III, n (%)	14 (35)	5 (55)	9 (30)	
IV, n (%)	5 (12)	3 (27)	2 (7)	
Abnormal ECG, n (%)	36 (90)	10 (100)	26 (87)	0.559
VT/VF, n (%)	7 (18)	3 (30)	4 (13)	0.361
Medications				
β-Blockers, n (%)	29 (73)	8 (80)	21 (70)	0.694
ACEI/ARB, n (%)	29 (73)	10 (100)	19 (63)	0.020
Aldosterone antagonists, n (%)	27 (68)	10 (100)	17 (57)	0.008
Loop diuretics, n (%)	22 (55)	9 (90)	13 (43)	0.011
Amiodarone, n (%)	2 (5)	2 (20)	0	0.067

Values are mean ± SD or n (%)

### CMR findings

The detailed CMR characteristics of children with LVNC are listed in Table 2. The mean LV ejection fraction (EF) and EDV index were 38.1 ± 17.4 % and 131.0 ± 55.8 mL/m^2^, respectively. No thrombus was detected in LV cavity on CMR. Non-compaction was present in 372 LV segments. The mean number of non-compacted segments per patient was 9.3 ± 2.5. The mean NC/C ratio was 3.64 ± 0.94.

**Table 2:** Cardiovascular magnetic resonance characteristics

	All Children	Presence of LGE		
Variable	(n = 40)	Yes (n = 10)	No (n = 30)	p Value
Heart rate (beats/min)	86.5 ± 22.8	92.3 ± 24.8	84.6 ± 22.2	0.360
Body surface area (m^2^)	1.6 ± 0.3	1.5 ± 0.2	1.6 ± 0.4	0.861
LV long-axis diameter (mm)	84.1 ± 12.0	86.1 ± 9.3	83.4 ± 12.8	0.534
LV Sphericity index	0.64 ± 0.21	0.75 ± 0.19	0.60 ± 0.20	0.045
LVEF (%)	38.1 ± 17.4	23.8 ± 10.7	42.9 ± 16.7	<0.001
LVEDV index (mL/m^2^)	131.0 ± 55.8	169.2 ± 65.1	118.2 ± 48.9	0.010
LVESV index (mL/m^2^)	87.8 ± 54.6	131.3 ± 55.5	73.3 ± 46.7	0.002
Stroke volume index (mL/m^2^)	43.2 ± 13.4	37.9 ± 17.0	44.9 ± 11.8	0.152
Myocardial mass index (mL/m^2^)	56.1 ± 19.0	64.6 ± 17.2	53.3 ± 19.0	0.107
Maximum NC/C ratio	3.64 ± 0.94	3.76 ± 1.24	3.60 ± 0.84	0.645
Number of non-compacted segments	9.3 ± 2.5	9.9 ± 2.8	9.1 ± 2.4	0.384
Duration of follow-up, years	3.0 ± 2.2	2.6 ± 2.3	3.2 ± 2.3	0.494

Values are mean ± SD

**Fig. 2:**
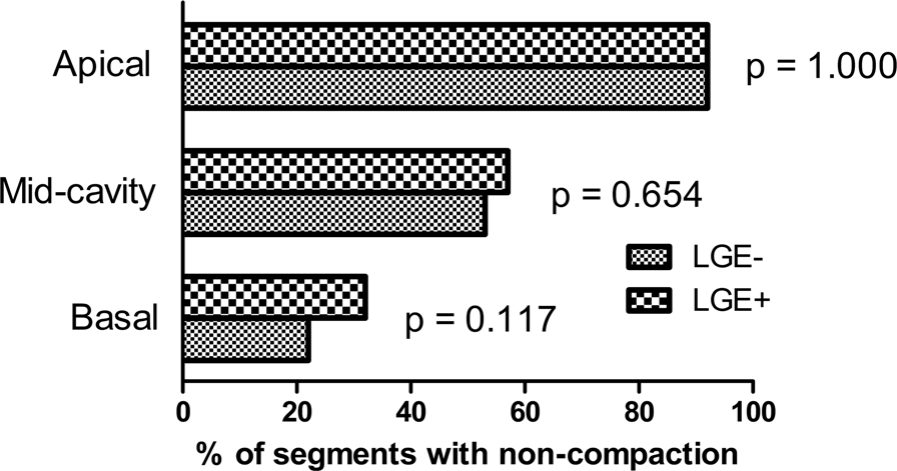
Anatomical distribution of non-compaction. Comparison of distribution of non-compaction according to region between LGE+ and LGE- children

A total of 10 (25 %) children with LVNC showed LV LGE. The LGE was observed in 46 segments, including 23 non-compacted segments and 23 normal segments. Of these, LGE was subendocardial in 11 segments, mid-wall in 18, subepicardial in 5, and transmural in 12. No association was found between myocardial layer distributions of LGE and LVEF (r = 0.49, p = 0.148). Compared LGE- children, LGE+ children had significantly lower LVEF (23.8 ± 10.7 % vs. 42.9 ± 16.7 %, p < 0.001) and greater LVEDV (169.2 ± 65.1 vs. 118.2 ± 48.9 mL/m^2^, p = 0.010), LV end-systolic volume (131.3 ± 55.5 vs. 73.3 ± 46.7 mL/m^2^, p = 0.002), and sphericity indices (0.75 ± 0.19 vs. 0.60 ± 0.20, p = 0.045). There were no differences in terms of number of non-compacted segments, NC/C ratio, and myocardial mass index between LGE+ and LGE- cohort. There was a similar distribution of non-compaction between LGE+ and LGE- children, which was more frequently observed on the apical segment than on the mid-cavity and basal segments (Fig. 2).

### Follow-up

**Fig. 3:**
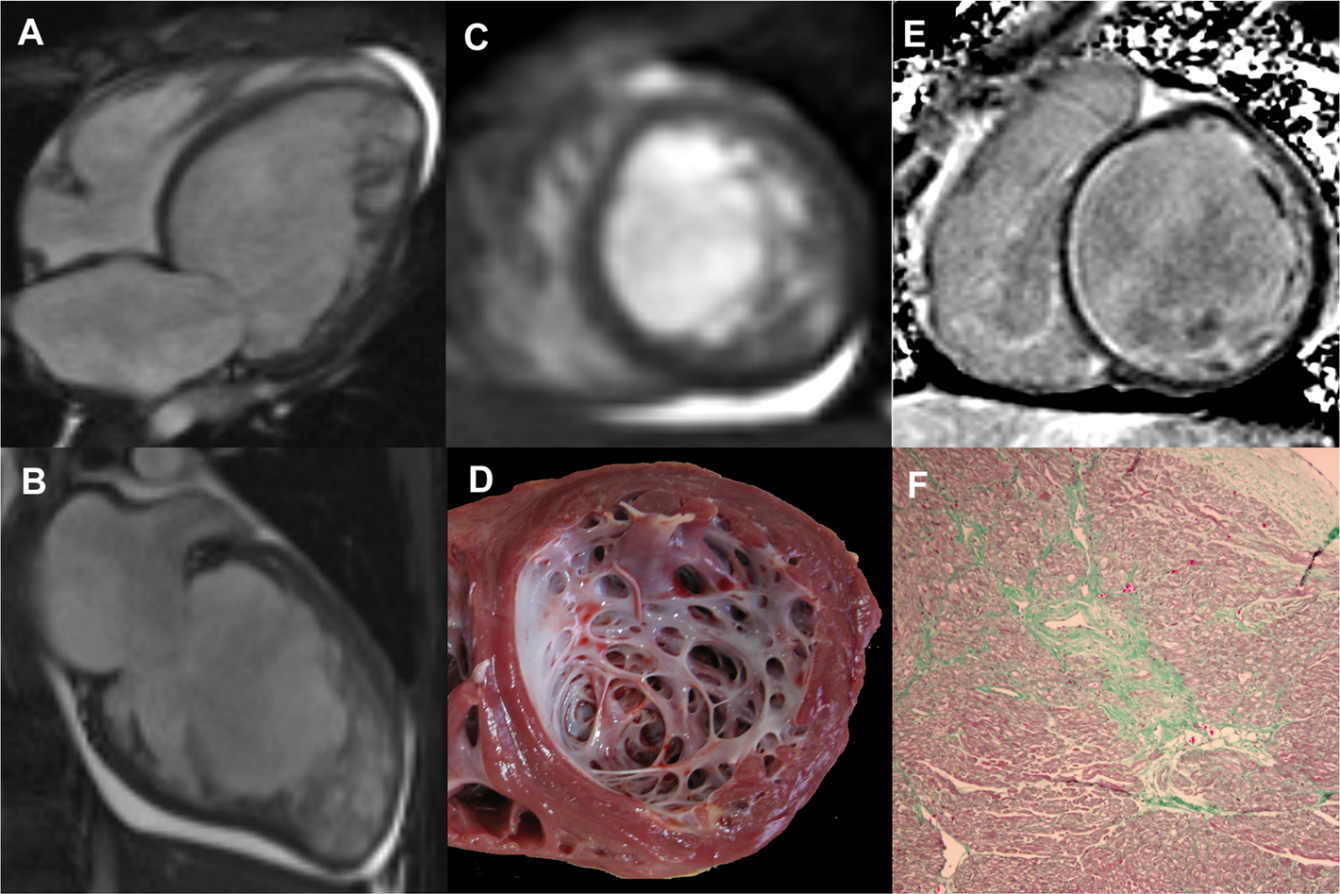
Representative images in a 14-year-old boy with LVNC underwent heart transplantation. The horizontal (**a**) and vertical (**b**) long-axis and short-axis (**c**) end-diastolic cine images showed prominent trabeculations in anterior, inferior, and lateral segments at apical level. The apical short-axis section of explanted heart (**d**) revealed prominent trabeculations. Contrast-enhanced image (**e**) demonstrated subendocardial LGE in the basal septum. Masson’s Trichrome staining (**f**) confirmed the present of replacement fibrosis in corresponding subendocardial area of basal septum (green area); magnification × 50

**Fig. 4:**
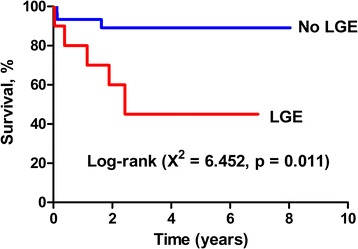
Adverse events and LGE in children with LVNC. Kaplan–Meier curve shows that the outcomes were worse in children with LGE

The average length of follow-up was 3.0 ± 2.2 years. During follow-up period, 6 children (15 %) died and 2 (5 %) underwent heart transplantation (Fig. 3), thereby resulting in a 20 % incidence of death and transplantation in the total cohort. There was no statistically significant difference in the length of follow-up between LGE+ and LGE- children (2.6 ± 2.3 versus 3.2 ± 2.3 years; p = 0.494). In LGE+ cohort, adverse events occurred in 6 children, including orthotropic heart transplantation in 2 children and death in 4 children. In LGE- cohort, adverse event was identified in 2 children. Kaplan-Meier analysis showed a significant difference in outcome between LGE+ and LGE- cohorts for cardiac death and heart transplantation (Fig. 4).

## Discussions

In the relatively large cohort study of children with isolated LVNC diagnosed by CMR, we investigated and compared CMR features and clinical outcomes between LGE+ and LGE- children. The overall occurrence of LGE (25 %) was lower to that found in previous adult studies [14, 15]. The LGE+ cohort exhibited a more maladaptive LV remodeling and higher incidence of cardiovascular death and heart transplantation.

CMR is showing increasing diagnostic potential in LVNC owing to a comprehensive identification and quantification of the extent of non-compacted myocardium [10, 20, 23]. The utility of CMR with LGE for detecting myocardial fibrosis is well established, which has also been confirmed in our previous report of the adult patient with LVNC [24]. Several studies have described a high prevalence of LGE in patients with LVNC, and LGE has been shown to be associated with ventricular arrhythmias as well as ventricular dysfunction [12–14]. Our previous study have shown that LGE was present in 19 (40 %) of the 47 patients and was more common in patients with ventricular arrhythmias by 24-h Holter electrocardiography recordings [12]. Dodd et al. [25] showed that the amount of trabecular LGE was correlated significantly with LVEF and an independent predictor of LVEF. Nucifora et al. [14] reported LGE was found in 55 % of patients and correlated with clinical severity and ventricular dysfunction. In keeping with their reports, our study showed LGE+ children had more severe LV dysfunction and NYHA functional class.

Similar to previous studies [12, 13], the distribution of LGE was strikingly heterogeneous and involved not only non-compacted segments but also normal segments in the present study. The exact cause of LGE in LVNC remains incompletely determined but is possibly associated with diminished coronary flow reserve, impaired microcirculatory function, coronary artery embolism, and maladaptive LV remodeling. Junga et al. [26] demonstrated restricted myocardial perfusion and decreased flow reserve in areas of ventricular non-compaction in children by positron emission tomography. They concluded the myocardial perfusion defects in non-compacted areas may be the cause of myocardial damage. Jenni et al. [27] found a decreased of coronary flow reserve is not confined to non-compacted segments, but extends to most segments with wall motion abnormalities, thus coronary microcirculatory dysfunction could be associated with ischemia and fibrosis in LVNC. Ridocci-Soriano et al. [28] and our previous studies [12] have demonstrated that coronary artery thrombo-embolism may possibly account for subendocardial or transmural LGE in patients with LVNC. In addition, the adverse cardiac remodeling caused by increased wall stress can result in focal cardiomyocyte necrosis due to augmentation of metabolic demands as well as coronary microcirculatory dysfunction. In the present study, the LV adverse remodeling was more severe in LGE+ children, as evidenced by higher LV volume and sphericity indices. Our study provided further evidence for the potential relationship between myocardial fibrosis and adverse remodeling.

Previous reports on pediatric LVNC associated with congenital heart lesions have suggested that mortality rates were from 15 % to 20 % during follow-up [6, 7]. Recent study of 242 children with LVNC by Brescia et al. [29] showed that 31 children (12.8 %) died and 13 (5.4 %) underwent heart transplantation, resulting in an 18.2 % incidence of death and transplantation in the median 4.0 years of follow-up. Similar to their studies, incidence of death and transplantation was 20 % in mean 3 years of follow-up in our children study cohort. Although the presence of LGE has been shown to be a strong discriminator of adverse outcomes in dilated and hypertrophic cardiomyopathies [30, 31], a very limited amount of data has been available on the prognostic value of LGE in children with LVNC. Our study showed a significantly higher rate of cardiac death and heart transplantation in LGE+ children. We propose that the presence of LGE could therefore potentially play an important role in stratification of treatment in children with LVNC. However, this finding should be interpreted with caution owing to the small sample size and lower occurrence of end-point events in the cohort. The pathophysiology and meaning of LGE in children with LVNC need to be elucidated in the future studies.

### Limitations

The present study has several limitations. First, the number of the cohort was still small owing to the relatively rare pediatric entity and a single-center nature. Second, there were potential selection and referral biases in our cohort, given most children clinically referred for CMR investigation had dramatic symptoms that may not be universally applicable to all populations. Third, LGE+ children had more adverse LV remodeling at baseline, which leads to indefinite relationship between the presence of LGE and adverse events. In order to conclusively ascertain incremental prognostic value of LGE in children with LVNC, larger studies with longer follow-up are required.

## Conclusions

In children with LVNC, the LGE is present in up to one-fourth of children with LVNC, and LGE+ children exhibited a more maladaptive LV remodeling and higher incidence of cardiovascular death and heart transplantation. The potential clinical utility of LGE in children with LVNC needs to be investigated by larger sample size studies with longer follow-up.

## Abbreviations

LVNC: Left ventricular non-compaction
CMR: Cardiovascular magnetic resonance
LV: Left ventricular
LGE: Late gadolinium enhancement
NC/C: Non-compacted-to-compacted
EDV: End-diastolic volume
ESV: End-systolic volume
LVEF: Left ventricular ejection fraction
NYHA: New York Heart Association
VT: Ventricular tachycardia
VF: Ventricular fibrillation
ACEI: Angiotensin-converting enzyme inhibitor
ARB: Angiotensin receptor blocker
